# Two-year follow-up of the COVID-19 pandemic in Mexico

**DOI:** 10.3389/fpubh.2022.1050673

**Published:** 2023-01-13

**Authors:** Antonio Loza, Rosa María Wong-Chew, María-Eugenia Jiménez-Corona, Selene Zárate, Susana López, Ricardo Ciria, Diego Palomares, Rodrigo García-López, Pavel Iša, Blanca Taboada, Mauricio Rosales, Celia Boukadida, Alfredo Herrera-Estrella, Nelly Selem Mojica, Xaira Rivera-Gutierrez, José Esteba Muñoz-Medina, Angel Gustavo Salas-Lais, Alejandro Sanchez-Flores, Joel Armando Vazquez-Perez, Carlos F. Arias, Rosa María Gutiérrez-Ríos

**Affiliations:** ^1^Departamento de Genética del Desarrollo y Fisiología Molecular, Instituto de Biotecnología, Universidad Nacional Autónoma de México, Cuernavaca, Morelos, Mexico; ^2^Facultad de Medicina, Laboratorio de Investigación en Enfermedades Infecciosas, División de Investigación, Universidad Nacional Autónoma de Mexico, Ciudad de México, Mexico; ^3^Departamento de Epidemiología, Instituto Nacional de Cardiología Ignacio Chávez, Ciudad de México, Mexico; ^4^Posgrado en Ciencias Genómicas, Universidad Autónoma de la Ciudad de México, Ciudad de México, Mexico; ^5^Departamento de Microbiología Molecular, Instituto de Biotecnología, Universidad Nacional Autónoma de México, Cuernavaca, Morelos, Mexico; ^6^Centro de Investigación en Enfermedades Infecciosas, Instituto Nacional de Enfermedades Respiratorias Ismael Cosío Villegas, Ciudad de México, Mexico; ^7^Centro de Investigación y de Estudios Avanzados del IPN, Laboratorio Nacional de Genómica para la Biodiversidad-Unidad de Genómica Avanzada, Irapuato, Guanajuato, Mexico; ^8^Centro de Ciencias Matemáticas, Universidad Nacional Autónoma de México, Morelia, Michoacan, Mexico; ^9^Coordinación de Calidad de Insumos y Laboratorios Especializados, Instituto Mexicano del Seguro Social, Ciudad de México, Mexico; ^10^Laboratorio Central de Epidemiología, Instituto Mexicano del Seguro Social, Ciudad de México, Mexico; ^11^Unidad Universitaria de Secuenciación Masiva y Bioinformática, Instituto de Biotecnología, Universidad Nacional Autónoma de México, Cuernavaca, Morelos, Mexico; ^12^Instituto Nacional de Enfermedades Respiratorias Ismael Cosío Villegas, Ciudad de México, Mexico

**Keywords:** COVID-19, variants, comorbidities, symptoms, logistic-regression, case-fatality-proportion

## Abstract

**Background:**

After the initial outbreak in China (December 2019), the World Health Organization declared COVID-19 a pandemic on March 11^th^, 2020. This paper aims to describe the first 2 years of the pandemic in Mexico.

**Design and methods:**

This is a population-based longitudinal study. We analyzed data from the national COVID-19 registry to describe the evolution of the pandemic in terms of the number of confirmed cases, hospitalizations, deaths and reported symptoms in relation to health policies and circulating variants. We also carried out logistic regression to investigate the major risk factors for disease severity.

**Results:**

From March 2020 to March 2022, the coronavirus disease 2019 (COVID-19) pandemic in Mexico underwent four epidemic waves. Out of 5,702,143 confirmed cases, 680,063 were hospitalized (11.9%), and 324,436 (5.7%) died. Even if there was no difference in susceptibility by gender, males had a higher risk of death (CFP: 7.3 vs. 4.2%) and hospital admission risk (HP: 14.4 vs. 9.5%). Severity increased with age. With respect to younger ages (0–17 years), the 60+ years or older group reached adjusted odds ratios of 9.63 in the case of admission and 53.05 (95% CI: 27.94–118.62) in the case of death. The presence of any comorbidity more than doubled the odds ratio, with hypertension-diabetes as the riskiest combination. While the wave peaks increased over time, the odds ratios for developing severe disease (waves 2, 3, and 4 to wave 1) decreased to 0.15 (95% CI: 0.12–0.18) in the fourth wave.

**Conclusion:**

The health policy promoted by the Mexican government decreased hospitalizations and deaths, particularly among older adults with the highest risk of admission and death. Comorbidities augment the risk of developing severe illness, which is shown to rise by double in the Mexican population, particularly for those reported with hypertension-diabetes. Factors such as the decrease in the severity of the SARS-CoV2 variants, changes in symptomatology, and advances in the management of patients, vaccination, and treatments influenced the decrease in mortality and hospitalizations.

## Introduction

In the last 2 months of 2019, cases of a novel severe pneumonia of unknown etiology were initially detected in the city of Wuhan, China. Using molecular biology techniques and genomic sequencing, its etiologic agent was characterized and classified as a new virus in the *Betacoronavirus* genus of the *Coronaviridae* family, phylogenetically closely related to severe acute respiratory syndrome coronavirus (SARS-CoV) and Middle East respiratory syndrome coronavirus (MERS-CoV). This new virus was designated severe acute respiratory syndrome coronavirus 2 (SARS-CoV-2) (1), and the associated disease was named coronavirus disease 2019 (COVID-19). After the initial outbreak in China and its subsequent epidemiological spread, the World Health Organization (WHO) declared COVID-19 a Public Health Emergency of International Concern on January 30^th^, 2020, and a pandemic on March 11^th^, 2020^1^ Before scaling to a global pandemic, the fatality rate in China ranged between 2 and 3.7% (2), with a greater impact observed among older adults. This rate pattern was reproduced around the world, where older adults were the most affected group (3). Additionally, reports indicated that a set of common symptoms associated with COVID-19, including fever, cough, dyspnea, sputum production, headache, myalgia, and fatigue, have been reported worldwide (4). Symptoms with a lower prevalence in the population, including diarrhea, hemoptysis and difficulty breathing (5), were also reported. Nevertheless, symptoms have varied, and anosmia and dysgeusia were also acknowledged as potential clinical markers of the disease. Different SARS-CoV-2 variants have emerged throughout the pandemic due to the natural accumulation of mutations in the viral genome that have circulated worldwide. To monitor viral evolution, the WHO encourages genomic analysis of virus samples. Relevant variants associated with a risk of global impact ranging from possible to alarming are classified as under monitoring (VUM), of interest (VOI), and of concern (VOC)^1^. Physicians treating COVID-19 patients have reported changes in the symptomatology associated with specific variants detected in the studied patients. For example, patients with the Omicron variant, first detected in samples collected in South Africa on November 14^th^, 2021, showed symptoms that were more similar to those of a common cold, mostly without anosmia or dysgeusia (6). In Mexico, the first SARS-CoV-2-positive sample was reported on February 27^th^, 2020, from a patient returning from a trip to Italy. By March 4^th^, the number of cases had risen to 80, strongly suggesting the onset of community transmission of the virus (7). On March 30^th^, 2020, the Mexican government emitted a national epidemiological alert placing the general population under lockdown and suspending non-essential activities, allowing only those related to health, security, governance, services, and the economy. By March 19^th^, 2022, there were more than 5.7 million confirmed cases and 324 thousand deaths according to official epidemiological reports from the Mexican Health Ministry (SSA for its acronym in Spanish, Secretaria de Salud) that monitored cases through the Respiratory Diseases Surveillance System (SISVER) and the General Directorate of Epidemiology (DGE). The SISVER database includes clinical and epidemiological information that allows the tracking of the pandemic^2^ Data available up to March 19^th^, 2022, show that the country has experienced several epidemiological surges (peaks or epidemiological waves) in the number of confirmed cases (CCs) of SARS-CoV-2 infection. During late 2020 and early 2021, Mexico registered a significant increase in the number of CCs, mainly driven by the B.1.1.519 variant (8), which was classified as a variant under monitoring (VUM) by the WHO in 2021. The vaccination campaign began for healthcare personnel and the over-60 population in the same period. Later, by June 2021, the pandemic was dominated by the Delta variant of concern (VOC) (9), while three other vaccines with different levels of effectiveness, as seen in Supplementary Table 1, were added to the vaccine campaign. Since December 2021, the Omicron variant and its sub-variants have been circulating in Mexico. This study aimed to detail the epidemiological evolution of the COVID-19 pandemic in Mexico during the first 2 years (March 2020–2022), the health policies adopted by the national government, and the circulating virus variants.

## Methods

### Settings

**Figure 1:**
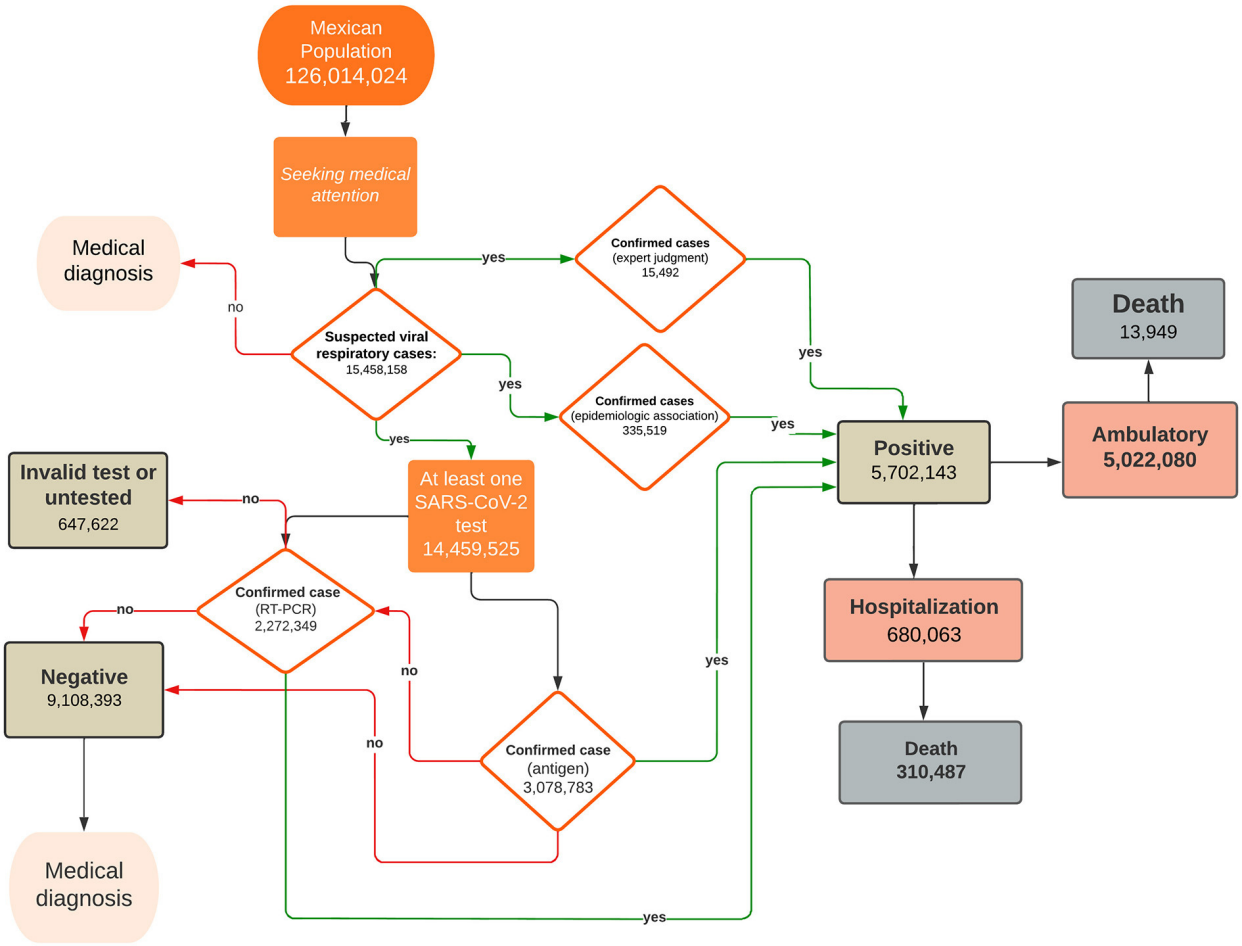
Follow-up of suspected COVID-19 cases reported in the SISVER database. The diagram shows suspected cases of viral respiratory disease and their evolution path. A fraction of the patients with a negative antigen test were tested by RT–PCR.

During the week of March 30^th^, 2020, the DGE declared a public health emergency and stated that the country had entered a community transmission stage. The strategy followed by the Mexican government for the epidemiological surveillance of COVID-19 is represented in Figure 1. The first step in the diagnosis began when a patient attended a health care unit after being in contact with an infected individual or showing symptoms. After filling out the admission form and questioning, the health personnel determined whether the patient should be tested. At the beginning of the pandemic, the patients were tested for SARS-CoV-2 only in hospitals. Then, the Mexican government published a standard that makes COVID-19 tests available to clinical laboratories and drugstores. Until mid-November, RT–PCR was the only test available, and antigen testing was authorized by Mexican health authorities. Six vaccines were used during the vaccine campaign in this period^3^, and the main characteristics and dates of approval are summarized in Supplementary Table 1.

#### Study population and design

This is a population-based longitudinal study.

#### Participants

All suspected COVID-19 cases recorded in the SISVER database.

#### Outcomes

Number of suspected cases (SCs), number of confirmed cases (CCs), number of CCs who died (deaths), number of CCs who were hospitalized (hospital admissions), case fatality proportion (CFP), and hospital admission proportion (HP).

#### Independent variable

Sex (men, women), age (0–17 yrs., 18–29 yrs., 30–39 yrs., 40–49 yrs., 50–59 yrs., 60+ yrs.), patient comorbidities (hypertension, obesity, diabetes, asthma, heart disease, renal insufficiency, COPD, immunosuppression, HIV/AIDS), patient symptoms, virus variant (lineages recorded in GSAID), and monthly percentages of vaccinated people.

#### Data source/measurement

The data provided by the SSA (through the DGE) contain suspected or confirmed cases, which include ambulatory, hospitalized, and deceased patients with demographic variables, self-reported comorbidities, and the main symptoms. **SCs** are patients seeking medical care as suspects (with symptoms or after contact with a CC). **CCs** are individuals with a quantitative reverse transcription polymerase chain reaction test (RT–PCR) positive for SARS-CoV-2, positive antigen tests or a positive result ruled by epidemiologic association (confirmed cases by epidemiologic association). **Confirmed cases by epidemiologic association** are SCs who have been in close contact (living within a distance of less than 1 meter for 15 continuous or cumulative minutes) with a laboratory-confirmed case by RT–PCR or rapid antigen test for SARS-CoV-2, from 2 to 14 days before the onset of symptoms and that the confirmed case to which it is associated, is registered on the SISVER platform or in the Online Notification System for Epidemiological Surveillance (SINOLAVE). **Deaths** are the CCs that held a death certificate. Hospital admissions are the CCs that were hospitalized. **CFP** is the fraction of deaths among the CCs. **HP** is the fraction of hospital admissions among the CCs. The operational definitions of outcomes were taken from the Mexican standard for epidemiological surveillance^4^ We chose the age groups following the vaccination strategy implemented by the Mexican government. The reported comorbidities were obtained through the suspected case study form completed during admission or health care visits. Patient symptoms were recorded by the health care personnel. For each suspected case tested, one or more tests can be conducted, but the data set in our study reports only the last result. Sequenced SARS-CoV-2 genomes from Mexico were uploaded to the GISAID database^5^ that had assigned lineage and date of complete sample collection (*n* = 47,572). Those sequences represent less than 1% of the CCs. Monthly percentages of people who received one dose of a vaccine and those who were fully vaccinated were downloaded from “Our World in Data”^6^

### Statistical analysis

The period analyzed in this work comprises epidemiological week 14 of 2020 (beginning on March 29^th^, 2020) up to epidemiological week 11 of 2022 (concluding on March 19^th^, 2022). Data were grouped according to the date of symptom onset. The distributions of the number of CCs, deaths and hospital admissions were analyzed by epidemiological period, sex, and age. Using the last result for every patient tested, we assessed a lower bound for the weekly number of tests (Supplementary Figure 2). The epidemic peaks were determined considering the changes in CC numbers in a three-week moving average of the weekly growth factor *G*_*n*_, where *n* means the nth week, which is calculated as the difference in natural logarithms (ln) of new cases accumulated in two subsequent weeks:


$$\begin{array}{l} G_{n}=\ln\left(N_{I\;}\left(t_{n}\right)\right)-\ln\left(N_{I\;}\left(t_{n-1}\right)\right) \end{array}$$

where *N*_*I*_(*t*_*n*_)are the new cases reported during week *n*th. This approach was chosen because, in the early stages of any epidemic, the number of infected patients grows exponentially at a given rate of *G* (10); this implies that the number of new infections in a time interval of length *t* is approximately expressed by I(*t*) α exp(*Gt*). Nevertheless, the weekly growth factor *G*_*n*_ is also helpful in obtaining information on contagion dynamics in every step of the pandemic. To characterize the waves, we performed descriptive analysis using simple frequencies and percentages of study variables. The CFP and HP were estimated as the average of 100 subsamples of size 15,000 taken from the original data set. After applying the Shapiro–Wilks test, we assumed the data's normality and calculated the 95% CI. To show the dynamics of the SARS-CoV-2 lineages that circulated in our country, a pile density curve was built. To show how symptoms have changed over time and how frequently they were among patients, we carried out cluster analysis. For the most frequent comorbidities among the Mexican population (diabetes, hypertension, and obesity), we also explored the combined effects of each pair of those comorbidities. Finally, we used multivariate logistic regression models with death and hospital admission as outcomes and the epidemic wave (1, 2, 3, 4), sex, age and the presence of comorbidities (yes, no) as risk factors. All analyses were performed with R v.4.1 statistical software^7^ We used ggplot2 v.3.3. to build the pile density curve and the *pheatmap* package^8^ with default options and the *complete* option to group symptoms throughout the study period.

## Results

**Figure 2:**
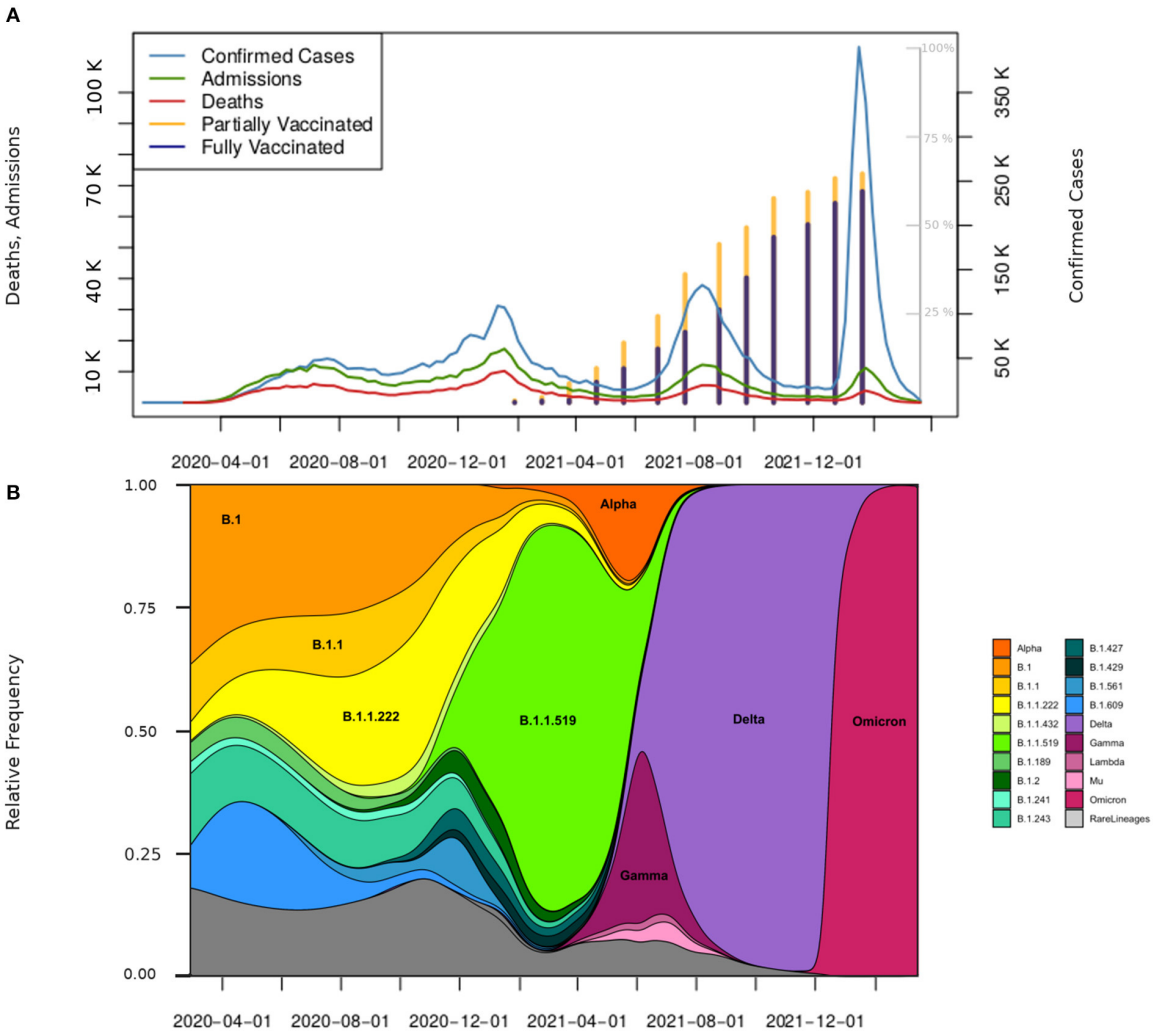
Confirmed cases, hospitalizations, deaths, vaccinations, and the presence of variants in Mexico. **(A)** Weekly distribution of CCs (scale on the right), hospitalizations, and deaths (scale on the left). The stacked bar plot in this figure presents the share of people partly (yellow bar) and fully vaccinated (navy bar). The percentage scale is displayed in gray on the inner right area of the plot. Bars are placed at the midpoint of the respective month beginning in January 2021. **(B)** Distribution of variants in the study period. The main variants that circulated in Mexico in the four evaluated periods are shown.

At the end of this study (March 19^th^, 2022), the national COVID-19 registry included a total of 15,458,158 suspected cases, out of which 5,702,143 were CCs, while 9,108,393 were not. The remaining 647,622 had no reported result because they were not tested or because the result was considered invalid (for example, due to poor sampling or poor handling of the test) (Figure 1). The overall fraction of CCs among the SCs (r_c_) is equal to 0.36, but it strongly varied over time and reached its maximum (0.67) in the last weeks of the study period. Supplementary Figure 1 shows that the peaks of r_c_ and epidemic waves approximately coincide. The curves of CCs, hospitalizations and deaths showed four peaks, but while those of CCs tended to increase, the others tended to decrease. The vaccination campaign started at the end of 2020, and in 1 year, 63.6% of the population had at least one dose (Figure 2A). Several virus variants became prevalent, with each time the latest replacing the previous one at a faster rate (Figure 2B). Of all CCs, 680,063 were hospitalized (11.9%), and 324,436 (5.7%) died. Even if the gender ratio of women to men was 1.08, males had a higher risk of death (CFP: 7.3 vs. 4.2%) and hospital admission risk (HP: 14.4 vs. 9.5%). Age was most strongly associated with the risk of death and admission. For the 60+ age group, CFP (26.8%) and HP (43.1%) were the highest; these values gradually decreased for the rest of the age groups. Remarkably, the HP curve by age is “J” shaped, with the 0–17 years group showing a higher HP (4.0%) than the next group.

**Figure 3:**
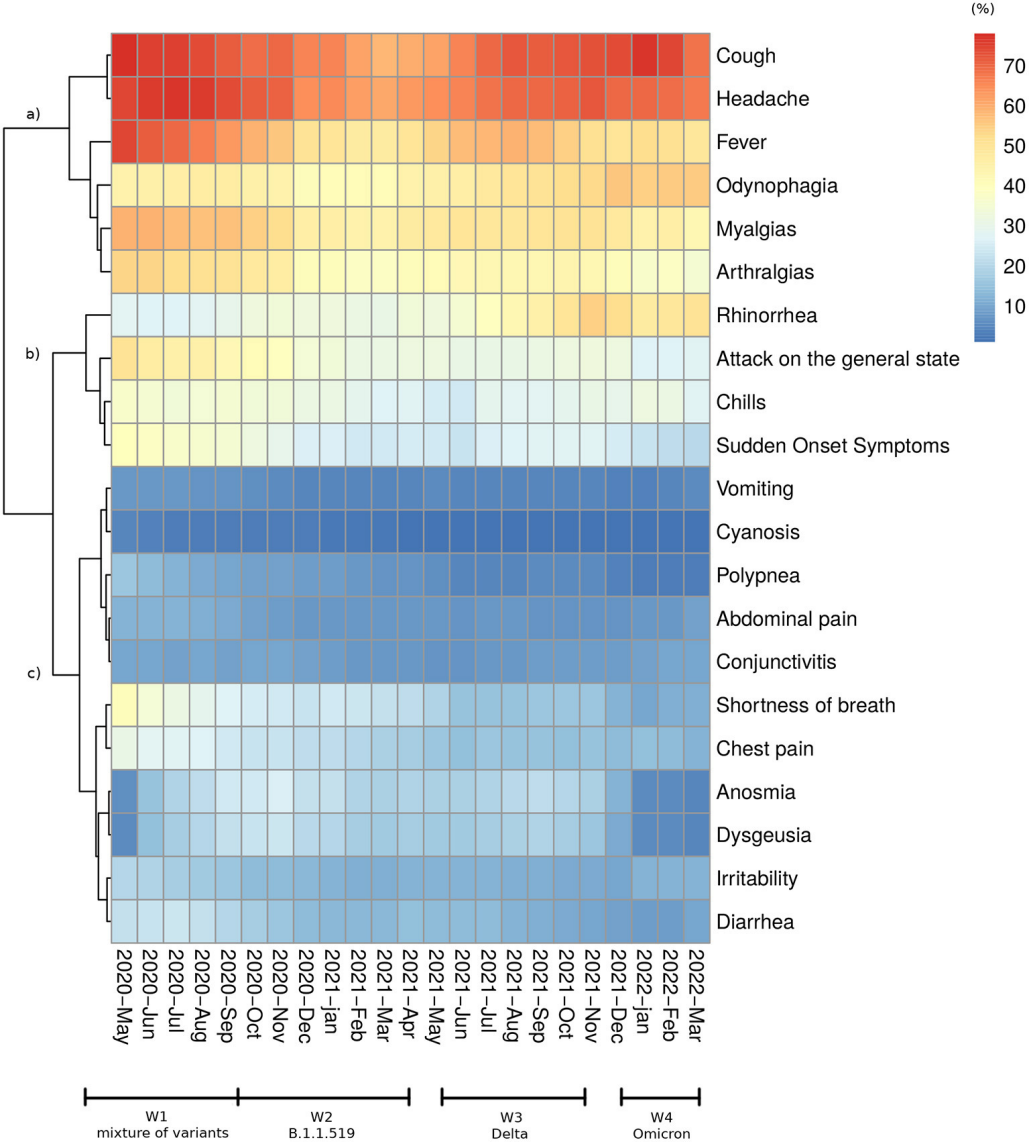
Symptom distribution. Heatmap and the cluster showing changes in the proportion of each recorded symptom among confirmed patients from May 2020 to March 2022.

Hypertension (12.7%), obesity (10.5%), and diabetes (9.5%) were the most prevalent comorbidities. Other comorbidities, such as heart disease, chronic obstructive pulmonary disease (COPD), renal insufficiency, and different immunosuppressive conditions, contributed to low percentages (<10%) among CCs. The percentage of deaths among people with diabetes was 21.9%, and that among people with hypertension was 19.8%, which was higher than the global percentage (14.5%). We also observed that the CFP of less prevalent comorbidities, such as renal insufficiency (38.1%), COPD (32.8%), and immunosuppression (21.6%), indicates an augmented risk of death (Table 1). In Figure 3, cluster (a) shows symptoms with a prevalence over 50% (cough, headache, fever, odynophagia, myalgias, and arthralgias); cluster (b) shows symptoms with a prevalence between 30 and 50% among people with CCs (rhinorrhea, chills, and sudden onset symptoms); and cluster (c) shows symptoms with a prevalence lower than 30% among people with CCs (vomiting, cyanosis, polypnea, abdominal pain, conjunctivitis, shortness of breath, chest pain, anosmia, dysgeusia, irritability, and diarrhea).

**Table 1:** Characteristics of COVID-19 infections, deaths, and hospitalization.

**Disease**	**Population** ^*****a*****^		**CCs**		**Deaths**		**Hospitalizations**	

	***n***	**%**	***n***	**%**	***n***	**CFP (%)**	***n***	**HP (%)**
**Gender**								
Male	61,473,390	(48.8)	2,734,533 (Median age 38.4 yrs.)	(48.0)	199,655 (Median age 63.7 yrs.)	(7.3)	395,354 (Median age 58.3 yrs.)	(14.5)
Female	64,540,634	(51.2)	2,967,610 (Median age 38.4 yrs.)	(52.0)	124,781 (Median age 65.0 yrs.)	(4.2)	284,709 (Median age 58.8 yrs.)	(9.6)
**Age (years)**								
0–17	38,521,344	(30.6)	369,277	(6.5)	1,262	(0.3)	14,822	(4.0)
18–29	24,729,112	(19.6)	1,395,260	(24.4)	5,418	(0.4)	35,030	(2.5)
30–39	18,441,103	(14.6)	1,295,787	(22.7)	15,767	(1.2)	63,795	(4.9)
40–49	16,445,999	(13.0)	1,106,120	(19.4)	37,089	(3.3)	104,002	(9.4)
50–59	12,733,490	(10.1)	793,746	(13.9)	66,048	(8.3)	142,813	(18.0)
60+	15,142,976	(12.0)	741,953	(13.0)	198,852	(26.8)	319,601	(43.1)
**Comorbidity**								
Hypertension	-	-	722,714	(12.7)	143,429	(19.8)	250,145	(34.6)
Diabetes	-	-	542,746	(9.5)	119,071	(21.9)	212,366	(39.1)
Obesity	-	-	599,034	(10.5)	67,261	(11.2)	132,498	(22.1)
Asthma	-	-	109,701	(1.9)	55,57	(5.1)	13,319	(12.1)
Heart disease	-	-	61,180	(1.1)	16,151	(26.4)	28,249	(46.1)
Renal insufficiency	-	-	60,275	(1.1)	22,986	(38.14)	37,618	(62.4)
COPD	-	-	42,917	(0.7)	14,084	(32.82)	23,377	(54.4)
Immuno-suppression	-	-	33,820	(0.6)	73,18	(21.64)	14,246	(42.1)
HIV/AIDS	-	-	16,594	(0.39)	1,506	(9.1)	3,297	(19.9)
Other	-	-	87,820	(1.5)	16,448	(18.7)	31,231	(35.6)
Overall^*^			1,403,710	(24.6)	203,104	(14.47)	380,477	(27.1)

Mexico March 2020-2022.

a Population census of 2020. “-” Unknown. “

* ” Refers to all comorbidities.

### Determining the waves

**Figure 4:**
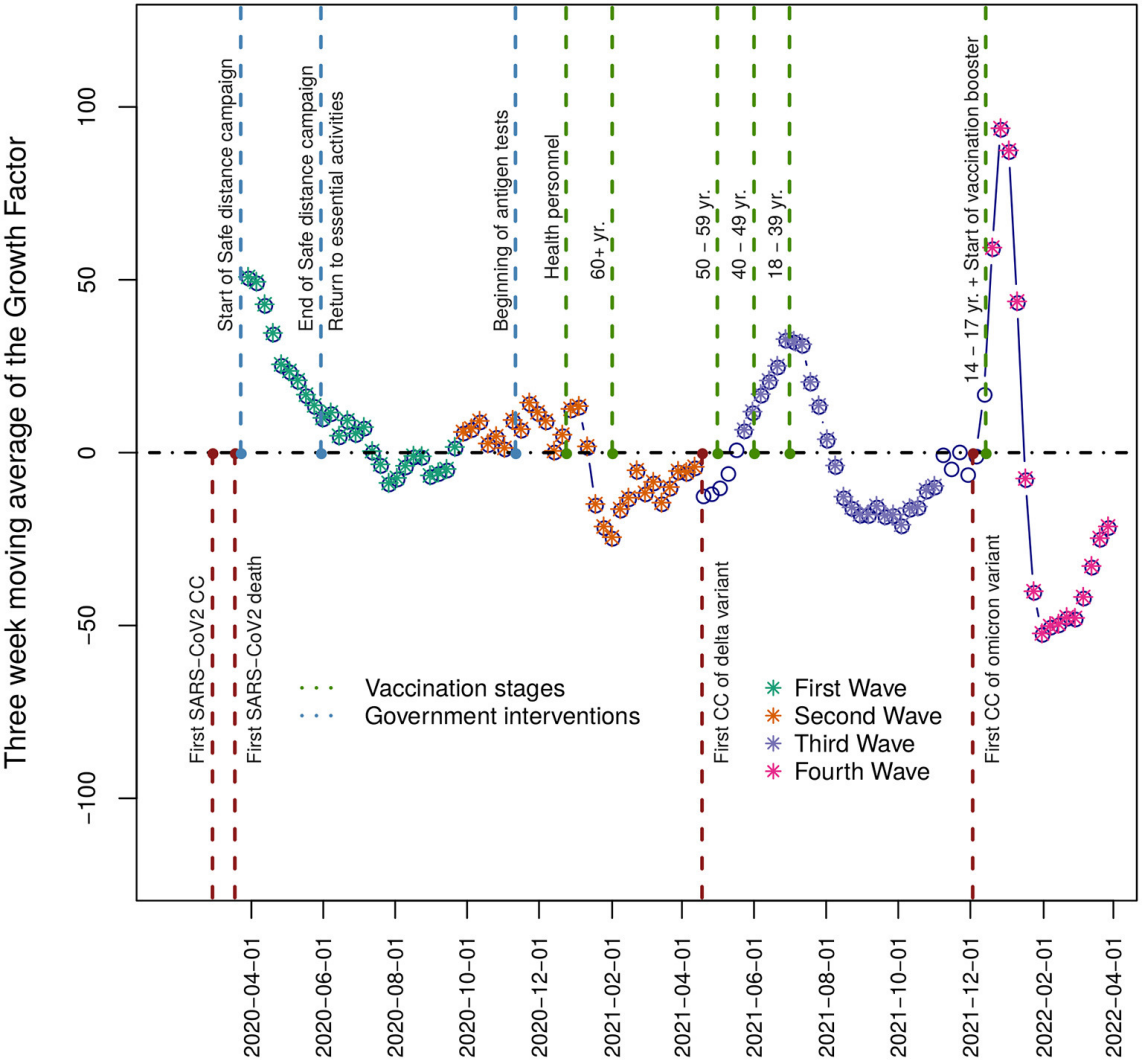
Growth Factor. The plot displays in the vertical axis the three-week moving average of the growth factor time series for the period under study—relevant events such as the progress in vaccination official strategy, variant detection, and public policy health interventions are shown. The filled points represent each wave, and the empty points represent the interwave periods.

We choose the date when the DGE declared the public health emergency (March 30^th^, 2020) as the starting day of the first wave. From that point, the weekly growth rate (*G*_*n*_) decreased from values that were over 50% to negative values (when the trend was inverted and began a brief period of decline in the number of CCs). After a period with *G*_*n*_≈0, there was a sudden increase at the beginning of week 40 (mid-September 2020) that marked the start of the second wave. The end of this second wave was followed by a five-week-long decline in CCs (0% > *G*_*n*_ > −12%). Afterward, as of epidemiological week 21 in 2021 (beginning on May 23^rd^, 2021), the beginning of the third epidemic wave was determined since *G*_*n*_ changed its sign. After a period of a moderate decrease at the end of the third wave (0>*G*_*n*_> −7%), a substantial increase was observed (*G*_*n*_≈100 %) in week 51 of 2021 (beginning on December 19^th^, 2021), becoming the fourth epidemiological wave. The *G*_*n*_value showed substantial variation throughout the examined waves. The relatively high values at the beginning of the pandemic constituted a transient phenomenon. Afterward, the maximum observed *G*_*n*_ value during the first and second epidemic waves was approximately 20%, rising from more than 30% in W3 to almost 100% during the fourth wave (Figure 4).

#### Wave 1 (W1)

The first wave started on March 29^th^, 2020 and ended on September 26^th^, 2020. During this period, 1,670,308 patients were tested at least once, and 809,387 CCs were detected with a median age of 43.7 years. Among CCs, there were 100,228 deaths (CFP = 12.3%) and 203,992 hospitalizations (HP = 25.1%) (Table 2). Approximately 150,000 CCs occurred in each 10-year age group over 18 and approximately 25,000 among children and adolescents (0–17 years). Hospitalizations and deaths increased exponentially with age (Supplementary Figure 3). The most common symptoms were headache, fever, myalgia, arthralgia, general malaise, and odynophagia (Figure 3). None of the circulating variants dominated during this first wave, and the most common variants included B.1, B.1.1, and B.1.1.222, from these, the first and the third variants reached its maximum prevalence (23%) in August-September 2020 (Figure 2B). The main strategy established by the Mexican government during this first wave was called the Safe Distance National Campaign, which began on March 23^rd^ and ended on May 30^th^, 2020 (Figure 4). After this measure, a remarkable decrease in weekly growth rate (*G*_*n*_) from values that were over 50% to values close to 20% (*G*_*n*_≲23%) was observed during mid-May. This *G*_*n*_ value remained positive and below 23% for 2 months until reaching its highest point in the 2^nd^ week of July 2020. At this turning point (*G*_*n*_ = 0), the trend was inverted and began a brief period of decline in the number of CCs followed by a (*G*_*n*_≈0) plateau.

**Table 2:** Epidemiological waves.

**Wave**	**Week starting**	Week^*^ **ending**	**Tested** ^**^ **(Tested per week)**	**Confirmed cases**	**Hospital admissions**	**Deaths**	**Case fatality proportion (95% CI)**	**Hospital admission proportion** ** (95% CI)**
**W1**	14 March 29^th^, 2020	40 September 26^th^, 2020	1,670,308 (61,863)	809,387	203,992	100,228	12.3% (10.8–13.7)	25.1% (23.0–27.1)
**W2**	41 September 27^th^, 2020	15 April 17^th^, 2021	4,302,882 (148,375)	1,538,110	251,245	132,638	8.7% (7.8–9.5)	16.4% (15.3–17.6)
**W3**	21 May 23^rd^, 2021	44 November 6^th^, 2021	4,289,906 (178,746)	1,439,463	140,266	61,155	4.2% (3.6–4.9)	9.8% (9.1–10.6)
**W4**	51 December 19^th^, 2021	11 March 19^th^, 2022	3,035,537 (202,369)	1,722,625	60,021	20,659	1.2%(0.9–1.5)	3.4% (2.8–4.0)

The four epidemic waves were established using the Gn value. The table shows the epidemiological weeks covered by each wave, start and end dates, and the number of confirmed cases, hospitalizations, and deaths, excluding the interwave periods. The CFP and HP values are the averages of 100 subsamples of size 15,000 taken from the original data set. After applying the Shapiro–Wilks test, we assumed the data's normality and calculated the 95% CI

* First week of each year: a) 2020: 2019-12-29; b) 2021: 2021-01-03; c) 2022-01-02. Weeks start on Sunday.

** Lower bound for the number of tests conducted.

#### Wave 2 (W2)

The second wave started in mid-September 2020 [when a new sustained increase in CCs was observed (Figure 2)] and ended in the 2^nd^ week of April 2021. In this period, 4,302,882 patients were tested, and 1,538,110 CCs were detected with a median age of 41.9 years. Among CCs, there were 132,638 deaths (CFP = 8.7%) and 705,673 hospitalizations (HP = 16.4%). With respect to W1, the overall proportions of deaths and hospitalizations were reduced by one-third (Table 2), while in all age classes, the number of CCs, hospitalizations and deaths increased (Supplementary Figure 3). The symptoms clusters a and b showed a steep decrease in prevalence (Figure 3). The initial prevalent variant B.1.1.222 was gradually replaced by the B.1.1.519 variant, and neither has been considered a VOC. During November 2020, the government authorized the use of antigen tests as a method to confirm the infection (Supplementary Figure 2), and a month later, it started the vaccination campaign. After the peak of January 2021, the number of weekly CCs decreased drastically. The increase in CCs was mainly associated with the dissemination of the B.1.1.519 variant (Figure 2B).

#### Wave 3 (W3)

The third wave began as of epidemiological week 21 in 2021 (beginning on May 23^rd^, 2021) and ended on November 6^th^, 2021. During this period, 4,289,906 patients were tested, and 1,439,463 CCs were detected with a median age of 34.7 years. Among CCs, there were 61,155 deaths (CFP = 4.2%) and 141,067 hospitalizations (HP = 9.8%). With respect to W2, the proportions of deaths and hospitalizations halved with respect to the previous wave (Table 2). CCs increased in individuals under 40 years of age, were stable in the 40–49 year age class and decreased in people aged 50 years of age and older, while deaths and hospital admissions declined in all ages and above all among older adults (Supplementary Figure 3). The data show an increase in rhinorrhea and odynophagia, such as a new increase in fever, cough, headache, and a decrease in shortness of breath and chest pain. Although Alpha and Gamma variants initially replaced the B.1.1.519 lineage, the Delta variant (appeared in June 2021) quickly became dominant (87% prevalence in August 2021) and characterized this wave (Figure 2B). The vaccination campaign progressed with the inclusion of individuals between 18 and 29 years of age. The maximum of this wave was followed by a decrease in CCs that ended in week 44 of 2021.

#### Wave 4 (W4)

On December 19^th^, 2021, the fourth epidemiological wave started (Figure 4). As of March 19, 2022, 3,035,537 patients were tested, and 1,722,625 CCs were detected with a median age of 36.5 years. Among CCs, there were 20,659 deaths (CFP = 1.2%) and 58,569 hospitalizations (HP = 3.4%). With respect to W3, the proportions of deaths and hospitalizations were reduced by two-thirds (Table 2), and CCs increased in adult age classes (18–59 years), while they slightly decreased in youngest (0–17 years) and older adults (60+ years). Deaths and hospital admissions continued to decline in all age groups (Supplementary Figure 3). The prevalence of anosmia and dysgeusia decreased. Infections in this wave were driven by the Omicron variant BA.1, which replaced the Delta variant very quickly (Figure 2B). The peak in weekly CCs was reached between the second and third week of January 2022 and lasted up to the fourth week of February 2022, when the CCs showed a steep fall (*G*_*n*_ <−50%) (Figure 4). The vaccination campaign included the first dose for individuals between 14 and 17 years and the booster shot for those aged 30 and over.

### Multivariable analysis of hospital admissions and deaths in Mexico during the 4 waves

Taking as reference the age group between 0 and 17 years, the group of 60 years or older reached a maximum in both admission and death risk with adjusted odds ratios of 9.63 (95% CI: 7.22, 13.11) and of 53.05 (95% CI: 27.94, 118.62), respectively. In both cases, the ORs follow a descending pattern (Tables 3, 4). Interestingly, for the age group of 18 to 29 years, and despite not being significant in the cases of deaths, our results for admissions OR = 0.52 (CI 95%: 0.37, 0.73) and for deaths OR = 0.95 (CI 95%: 0.46, 2.25) imply a reduction in the admission and death risk concerning the reference group. We also observed a progressive reduction in the admission and death risk as the four waves elapsed, with the fourth wave displaying a stronger association with the decrease in admission and death risks. For this wave (W4), the fitted values for ORs in the admissions and deaths case were equal to 0.15 (95% CI: 0.13–0.18). Overall, men had higher odds of admissions (OR = 1.59; 95% CI: 1.44, 1.75) and deaths (OR = 1.78; 95% CI: 1.60, 1.97). Finally, the presence of comorbidities was associated with an increased admission (OR = 2.40; 95% CI: 2.17, 2.66) and death (OR=2.38; 95% CI: 2.14, 2.66) risk. For each combination of hypertension, diabetes and obesity, we observed, throughout the waves, a downward trend in the percentage of COVID-19 patients with those comorbidities (Supplementary Figure 5). We can note a similar behavior in the case of deaths, except for the group of patients with both diabetes and hypertension, for which we observe an increased contribution to the total deaths in the fourth wave. We also note that the proportion of all these comorbidities in the CC increases by at least a factor of two in the total number of deaths for all the waves.

**Table 3:** Hospital admissions risk.

	**Odd ratio**	**95% CI**	***P* value**
**Wave**			
Wave 4	0.15	0.12 to 0.18	<2e-16
Wave 3	0.52	0.45 to 0.60	1.8E-13
Wave 2	0.72	0.64 to 0.81	1.0E-06
Wave 1	1.0	-	-
**Sex**			
Men	1.78	1.60 to 1.97	<2e-16
Women	1.0	-	-
**Age**			
> 59 yr.	53.05	27.94 to 118.62	< 2e-16
50–59 yr.	15.35	8.05 to 34.43	6.6E-11
40–49 yr.	6.69	3.49 to 15.06	1.5E-06
30–39 yr.	2.67	1.37 to 6.08	0.0105
18–29 yr.	0.95	0.46 to 2.26	0.9004
0–17 yr.	1.0	-	-
**Comorbidities**			
Yes	2.38	2.14 to 2.66	< 2e-16
No	1.0	-	-

Multivariable logistic regression results for risk of hospital admission among patients with coronavirus disease.

**Table 4:** Death risk.

	**Odd ratio**	**95% CI**	***P* value**
**Wave**			
Wave 4	0.15	0.13 to 0.18	< 2e-16
Wave 3	0.46	0.40 to 0.53	< 2e-16
Wave 2	0.59	0.52 to 0.66	5.9E-13
Wave 1	1.0	-	-
**Sex**			
Men	1.59	1.44 to 1.75	1.9E-14
Women	1.0	-	-
**Age**			
>59 yr.	9.63	7.22 to 13.11	< 2e-16
50–59 yr.	3.18	2.37 to 4.35	6.0E-11
40–49 yr.	1.68	1.25 to 2.30	0.001275
30–39 yr.	0.93	0.68 to 1.28	0.630152
18–29 yr.	0.52	0.37 to 0.73	0.000205
0–17 yr.	1.0	-	-
**Comorbidities**			
Yes	2.40	2.17 to 2.66	< 2e-16
No	1.0	-	-

Multivariable logistic regression results for risk of death among patients with coronavirus disease.

## Discussion

From the beginning of the pandemic to March 29^th^, 2022, there were a total of 490,204,256 confirmed cases and 6,173,572 deaths around the world^9^ The highest number of infections favored the appearance of new variants with some evolutionary advantage. The local emergence and dominance of SARS-CoV-2 variants as well as the health system responses modeled the pattern of the pandemic in the COVID-19 epidemiological profiles of countries (11). In Mexico, the first case of COVID-19 was recorded on February 27, 2020. For almost 1 month, the detected infections were all imported. The first local transmissions were reported on March 23^rd^, 2020, 1 week later, when the government declared a public health emergency (12); since then, and until March 2022, four epidemic waves have occurred, in contrast to Italy, where five waves and VOCs were reported (11).

### Virus spread and evolution

As in the rest of the world, at the onset of the pandemic, a patchwork of virus variants circulated in Mexico (7). In the second wave, B.1.1.519 was the dominant variant. Rodriguez-Maldonado et al. (8) reported a sequence mutation at position T478K in the S protein (8) that may be involved in immune evasion and transmission advantage over the previous circulating variants. At the end of W2, the Alpha variant appeared first and spread faster than B.1.1.519, followed by Gamma, which spread even faster in some areas of the country (13). Finally, the introduction of the Delta variant occurred during mid-June 2021 (9), which reached 87% prevalence in August 2021 during the third wave peak (Figure 2B). In the fourth wave and as of December 2021, the Omicron variant (BA. 1) pushed out the Delta variant and became the most prevalent in March 2022 (the end of this analysis), representing over 90% of the sequences obtained (Figure 2B). As reported in several studies, all the VOCs showed each time an increased transmissibility with respect to the previous one (14). Even if the distribution of CCs over time also depends on the health policies adopted at the national and local levels, such as on the behavior of the population, the dynamic of the CCs from W2 to W4 (associated with the prevalent variants) is consistent with the ever-greater spread capacity developed by the variants that have followed one another. The evolution of the virus also altered patients' manifested symptomatology. At the onset of the pandemic (4), the most frequent symptoms were “similar to that of an acute respiratory infection”, such as headache, fever, myalgia, arthralgia, general malaise, and odynophagia (Figure 3). Since anosmia and dysgeusia were poorly associated with other coronaviruses, these symptoms were not considered for diagnosis in the surveillance of W1, thus hindering early detection and treatment. The progression of the pandemic and of cases of the B.1.1.519 variant showed a decrease in symptoms such as cough and headaches. In line with results from other studies (15), symptoms such as rhinorrhea and odynophagia were more prevalent with the Delta variant. Instead, cough, fever, myalgia, malaise, headache, body ache, and moderate to severe fatigue were more common with Omicron (W4), supporting the assumption that this variant infects mainly the upper respiratory tract (16). Our data also confirmed (Figure 3) that anosmia was less prevalent in Omicron infections (17) and indicate that diagnosis is a challenge to physicians as new variants emerge.

### Health policies and health system response

The maximum value recorded in the first wave for the growth factor *G*_*n*_ was the lowest for the four waves. This behavior could be related to the Safe Distance National Campaign proposed by the Mexican government on March 23^rd^, 2020. The campaign included school lockdowns and reduced economic activities, retaining only essential services. However, the were a relative limited number of diagnostic tests, thus reducing the detection of cases. Furthermore, the results of the logistic regression show that this wave presents, globally, both the highest admission and death risk. This behavior is confirmed in a study showing the leading causes of excess mortality in Mexico during 2020–2021 (18) and suggests that the safe distance campaign was a useful measure to reduce the number of CCs but had less impact on the proportion of hospitalized and deceased patients. W2 showed an increase in CCs compared with W1 (Figure 2), partly due to the higher number of total infections and the improved detection of infections. On the one hand, fewer restrictions on population mobility increased the contact rate. On the other hand, the introduction of rapid tests increased the total number of tests conducted daily and allowed for more comprehensive monitoring of the pandemic. As shown in Table 2, W2 is also characterized by the highest number of deaths and hospital admissions and by the highest ratio of deaths/admissions (>0.5). Several reasons may have contributed to this result, such as hospital saturation and high occupancy of intensive care units in public and private institutions, as well as an increase in in-house oxygen demand. In W3, Delta dominance caused an increase in infections among younger ages that were not yet (0–17 years) or were just (18–29 years) included in the vaccination campaign. In contrast, infections among individuals over 50 years of age (the first to complete the vaccine cycle) decreased. Even if several studies found that Delta was the most virulent VOC (19), there was a decrease in hospital admissions and deaths that can be explained by several factors. One of them is the progress in the vaccination campaign, which for the first time included individuals between 18 and 59 years. By the end of July 2021, 16 and 20% of the total population were partially and fully vaccinated, respectively. In this period, other vaccines were introduced in the vaccine campaign with differences in effectiveness (Supplementary Table 1); however, deaths and hospitalizations continued to decline, indicating that this vaccine mosaic gave reasonable protection in the Mexican population and decreased the severity of the registered cases. Nonetheless, another factor that helped to reduce the number of deaths as the pandemic continued was the acquisition of knowledge in treating the disease by health professionals. At the beginning of the pandemic, authorities recommended staying home until symptoms such as fever or chills and shortness of breath appeared. Currently, the recommendation is to receive health care if someone is suspected to be infected with SARS-CoV-2. This last recommendation leads to a better diagnosis and early treatment. Additionally, the introduction of antivirals and steroids, known for preventing progression to respiratory failure and death (20), were important factors in decreasing the death rate. It is important to highlight that a better treatment regimen in light of the molecular evolution of the virus has altered how the immune system faces the disease (21). In the fourth wave, the higher exposure of individuals to the new and more transmissible Omicron variant (due to the resumption of social activities) may have caused the observed upturn of CCs in the 18–49 age group. Additionally, in the case of the 50–59 age group, the loss of vaccine effectiveness caused by a decline in neutralizing antibodies against SARS-CoV-2 (22) may have been another reason for the increase in CCs, since these groups received the first two doses between May and July of 2021. Interestingly, W4 presented a decline in the frequency of CCs with comorbidities compared with the first wave (Supplementary Figure 4). This result can be associated with advances in vaccination and changes in the severity of the illness.

### Fragile population

Worldwide reports have shown that while both sexes show the same susceptibility to COVID-19 infection, males belong to the population most vulnerable to COVID-19. Furthermore, aging and underlying comorbidities represent two serious risk factors for developing severe disease (23, 24). Consistent with those findings, in Mexico, while there was no difference in the likelihood of becoming infected between sexes (Table 1), males had higher odds than females of being hospitalized and dying (Tables 3, 4), and patients older than 50 years showed the highest odds of being hospitalized or dying compared to younger people (0–29 years). The presence of comorbidities also represented an important risk factor for the development of severe infection, increasing the odds of hospitalization and death by almost 2.5 times (Tables 3, 4). The highest risks of hospitalizations and deaths observed, especially in the first and second waves, could be related to the high prevalence of obesity, diabetes, and hypertension in all age groups of the Mexican population (25). In 2016, Mexico declared an obesity health emergency, where 76.0% of adults were overweight and obese^10^ In 2020, the Health and Nutrition National Survey (ENSANUT as its acronym in Spanish) reported a diabetes prevalence of 15.7% and a prevalence of hypertension of 30.2% among people over 20 years of age^11^ The combination of hypertension and diabetes strongly compromises the prognosis of COVID-19 patients. These conditions combined with aging represented the higher risk of death among those included in our study.

### Limitations and strengths of the study

Especially in the first wave, the data suffer from bias due to undetected patients. However, the introduction of rapid testing allowed more complete monitoring of the pandemic. Nevertheless, the data are a highly reliable and complete source of information on the health strategy followed by the Mexican government. The experience of the first 2 years of the pandemic could help to define health policies for the follow-up of future epidemics and pandemics. It would be advisable to include the establishment of active contact system tracing in the national pandemic plan and defining a minimum threshold for the number of intensive care units at the regional level based on the population age, health and density. The information delivered by these data and their analysis could provide the general population with educational tools and access to health care services that improve their quality of life and allow them to face this and subsequent epidemics as a healthy and informed population.

## Data availability statement

The raw data supporting the conclusions of this article will be made available by the authors, without undue reservation.

## Author contributions

AL, RW-C, M-EJ-C, and RG-R conceived and designed the methodology and analysis. RC, DP, and SZ contributed to data processing. AL and RG-R wrote the manuscript. AL, SZ, and RG-R prepared the figures and tables. RW-C, M-EJ-C, SZ, SL, RC, DP, RG-L, PI, BT, MR, CB, AH-E, NM, XR-G, JM-M, AS-L, AS-F, and JV-P reviewed the final version of the manuscript. CA helped improve the project, coordinated genomic surveillance, and reviewed the final version of the manuscript. RG-R coordinates the epidemiologic analysis group. All authors contributed to the article and approved the submitted version.

## Footnotes

^1^ https://www.who.int/en/news/item/27-04-2020-who-timeline—covid-19

^2^ https://coronavirus.gob.mx/informacion-accesible/

^3^ https://covid19.trackvaccines.org/country/mexico/

^4^ https://coronavirus.gob.mx/wp-content/uploads/2022/01/2022.01.12-Lineamiento_VE_ERV_DGE.pdf

^5^ https://gisaid.org/hcov19-variants/

^6^ https://ourworldindata.org/grapher/covid-vaccination-doses-per-capita?country~MEX

^7^ https://cran.r-project.org/

^8^ https://CRAN.R-project.org/package=pheatmap

^9^ https://covid19.who.int/

^10^ https://www.gob.mx/salud/prensa/emite-la-secretaria-de-salud-emergencia-epidemiologica-por-diabetes-mellitus-y-obesidad

^11^ https://ensanut.insp.mx
