# Supplementary material for: Two-year follow-up of the COVID-19 pandemic in Mexico
Source: Front Public Health. 2023 Jan 13;10:1050673. doi: 10.3389/fpubh.2022.1050673 (PMC9880891; doi:10.3389/fpubh.2022.1050673)
Supplement: Supplementary file 1 [file Data_Sheet_1.PDF]

Supplementary Material

Table 1 SM: Main characteristics of vaccines used in the COVID-19 vaccine campaign in Mexico to March 2022.

**Characteristic**	**Vaccine**					
	**BNT162b2**[1]	**ChAdOx1-S**[1]	**Sputnik V**[1]	**Ad26.COV2. S**[1]	**CoronaVac**[1]	**Ad5-nCoV**[2]
**Developer**	Pfizer-BioNTech	University of Oxford and AstraZeneca	Gamaleya	Janssen and Jhoson & Jhoson	Sinovac Biotech	CanSino
**Type of vaccine**	mRNA[3]	Chimpanzee adenoviral vector	Human adenoviral vectors	Human adenoviral vector	Inactivated virus	adenovirus type 5 vector
**Dosage**	Two 21 days apart [4]	Twelve days appart[5,6]	Two 21 days apart[7]	One[8]	Two 14 days apart[9]	One[2]
**Efficacy**	95%	81.3%	91.6%	66%	50.7%	63.7%
**Side effects**	Local post-injection pain, fatigue, headache anaphylaxis, and myocarditis	Local post-injection pain, fever, and headache	Local post-injection pain, hyperthermia, asthenia, headache, muscle, and joint pain.	Local post-injection site pain, headache fatigue, muscle pain, and nausea.	Local post-injection site pain, headache fatigue.	Pain, itching, swelling, and redness at the injection site, tiredness, headache, muscle and joint pain, fever, diarrhea, nausea, vomiting, low appetite, dizziness, cough, and sore throat [10]
**Authorized in Mexico.**	December 11, 2020[10]	January 04, 2021[10]	February 02, 2021[10]	May 27, 2021[10]	February 09, 2021[10]	February 09, 2021[10]

[1] Hadj Hassine, I., 2022. Covid-19 vaccines and variants of concern: a review. Reviews in medical virology, 32(4), p.e2313.

[2] Halperin, S.A., Ye, L., MacKinnon-Cameron, D., Smith, B., Cahn, P.E., Ruiz-Palacios, G.M., Ikram, A., Lanas, F., Guerrero, M.L., Navarro, S.R.M. and Sued, O., 2022. Final efficacy analysis, interim safety analysis, and immunogenicity of a single dose of recombinant novel coronavirus vaccine (adenovirus type 5 vector) in adults 18 years and older: an international, multicentre, randomised, double-blinded, placebo-controlled phase 3 trial. The Lancet, 399(10321), pp.237-248.

[3] Unlike vaccines that put a inactivated germ into the body, the mRNA vaccine delivers a tiny piece of genetic code from the SARS CoV-2 virus giving cells instructions, for making copies of spike proteins. The spikes do the work of penetrating and infecting host cells. These proteins stimulate the production of antibodies.

[4] Walsh EE, Frenck RW, Falsey AR, et al. Safety and immunogenicity of two RNA-based covid-19 vaccine candidates. N Engl J Med. 2020;383(25):2439-2450.

[5] Folegatti PM, Ewer KJ, Aley PK, et al. Safety and immunogenicity of the ChAdOx1 nCoV-19 vaccine against SARS-CoV-2: a preliminary report of a phase 1/2, single-blind, randomised controlled trial. Lancet. 2020;396(10249):467-478. https://doi.org/10.1016/ S0140-6736(20)31604-4

[6] Voysey M, Costa Clemens SA, Madhi SA, et al. Single-dose administration and the influence of the timing of the booster dose on immunogenicity and efficacy of ChAdOx1 nCoV-19 (AZD1222) vaccine: a pooled analysis of four randomised trials. Lancet. 2021;397(10277):881-891. https://doi.org/10.1016/ S0140-6736(21)00432-3.Balakrishnan VS. The arrival of Sputnik V. *Lancet Infect Dis*. 2020; 20(10):1128. https://doi.org/10.1016/S1473-3099(20)30709-X

[7] Balakrishnan VS. The arrival of Sputnik V. Lancet Infect Dis. 2020; 20(10):1128. https://doi.org/10.1016/S1473-3099(20)30709-X

[8] Sadoff J, Gray G, Vandebosch A, et al. Safety and efficacy of single- dose Ad26.COV2.S vaccine against covid-19. N Engl J Med. 2021; 384(23):2187-2201. https://doi.org/10.1056/nejmoa2101544

[9]Schillie S, Harris A, Link-Gelles R, Romero J, Ward J, Nelson N. Recommendations of the Advisory Committee on Immunization Practices for use of a hepatitis b vaccine with a novel adjuvant. MMWR Morb Mortal Wkly Rep. 2018;67(15):455-458. https://doi. org/10.15585/mmwr.mm6715a5 [x]

[10] https://vacunacovid.gob.mx/informacion-de-la-vacuna/

Supplementary figures.

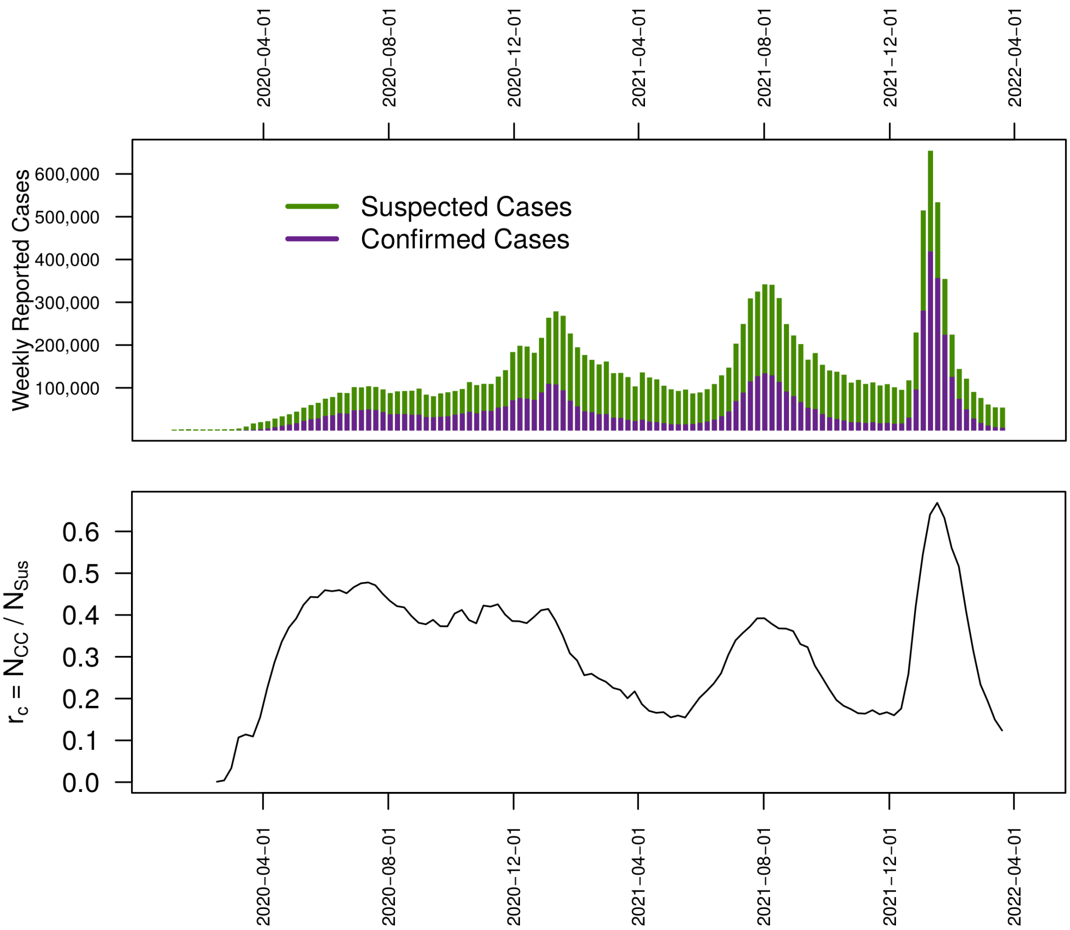

Figure 1 SM: Suspected and confirmed cases. The plot shows the number of suspected and confirmed cases reported in the SISVER database (top plot). We also show (bottom plot) the proportion of CCs calculated with the formula $r_c=N_{cc}/N_{sus}$, where N_{cc} and N_{sus} are the number of CCs and suspected cases in each week, respectively.

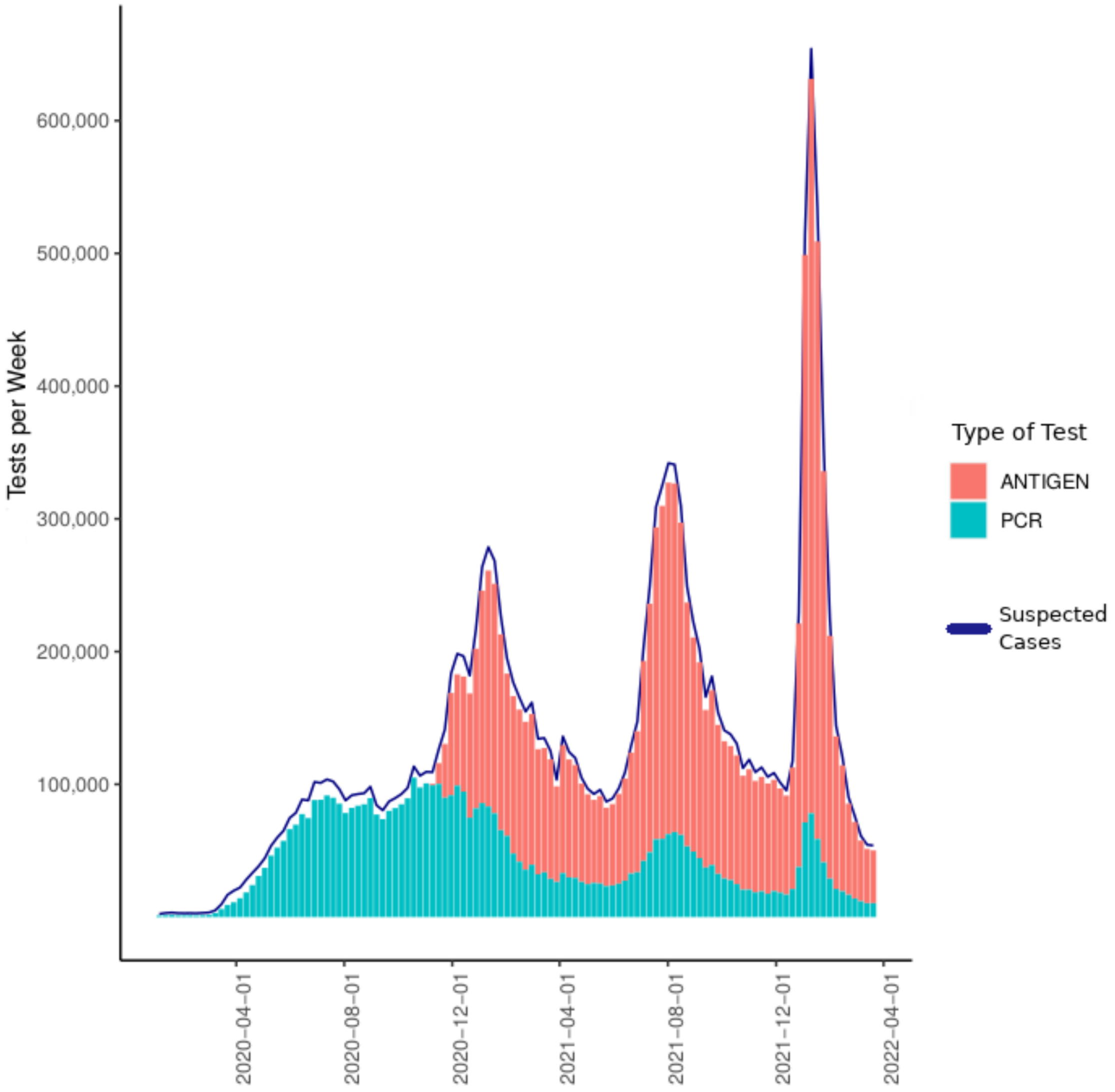

Figure 2 SM: Number of tests conducted over suspects for SARS-CoV-2 in Mexico. The plot represents a lower bound for the number of SARS-CoV-2 tests, antigens and RT–PCR conducted per week in persons seeking medical assistance (stacked bar plot). We also show, for reference, the weekly number of suspected cases (dark blue solid line).

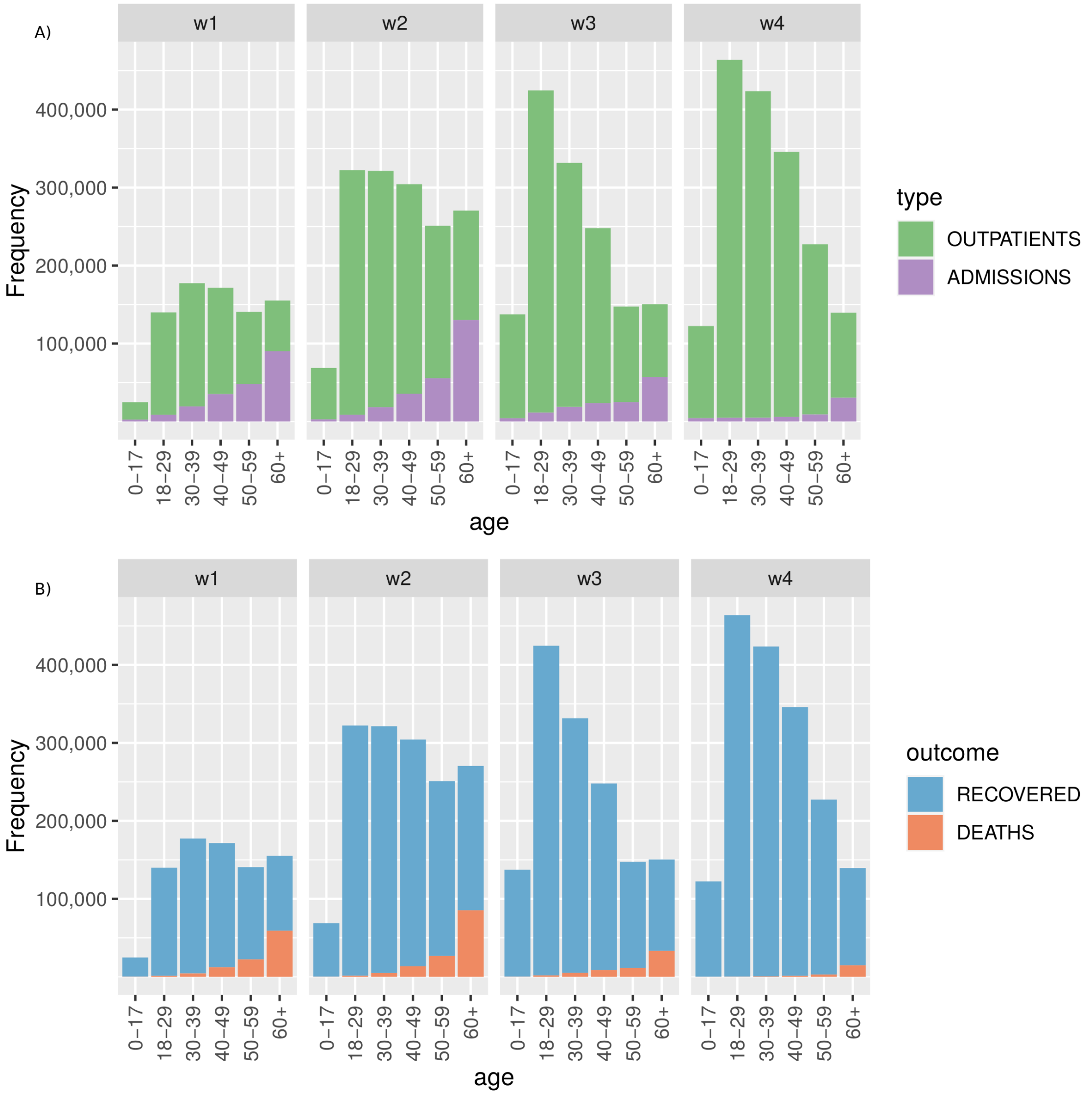

Figure 3 SM. Patterns of death, hospitalizations, outpatient visits and recovered cases by age group. A) Outpatients and hospitalized patients by age group. The distribution of outpatients and hospitalized patients by age group in the four periods is shown. B) Recovered patients and deaths by age group. The distribution of recovered patients and deaths by age group in the four periods is shown. The age intervals in both plots are grouped by the age groups considered for vaccination by the official strategy in Mexico.

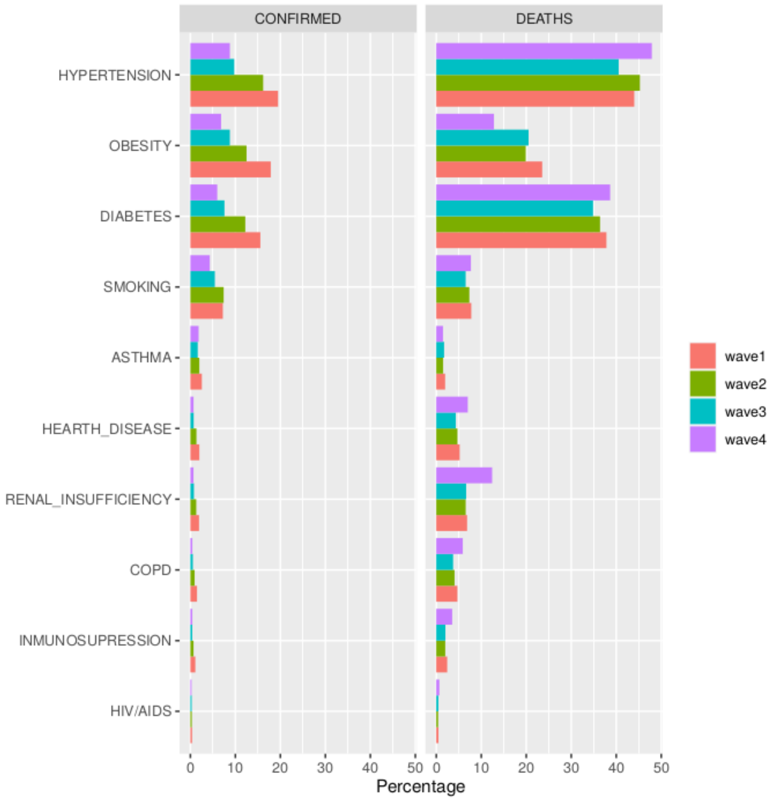

Figure 4 SM. Distribution of comorbidities and conditions by wave. Confirmed cases (left panel). Distribution of percentages of patients with comorbidities and conditions in each period. Confirmed deaths (right panel). Distribution of the percentages of deceased patients with comorbidities in each period.

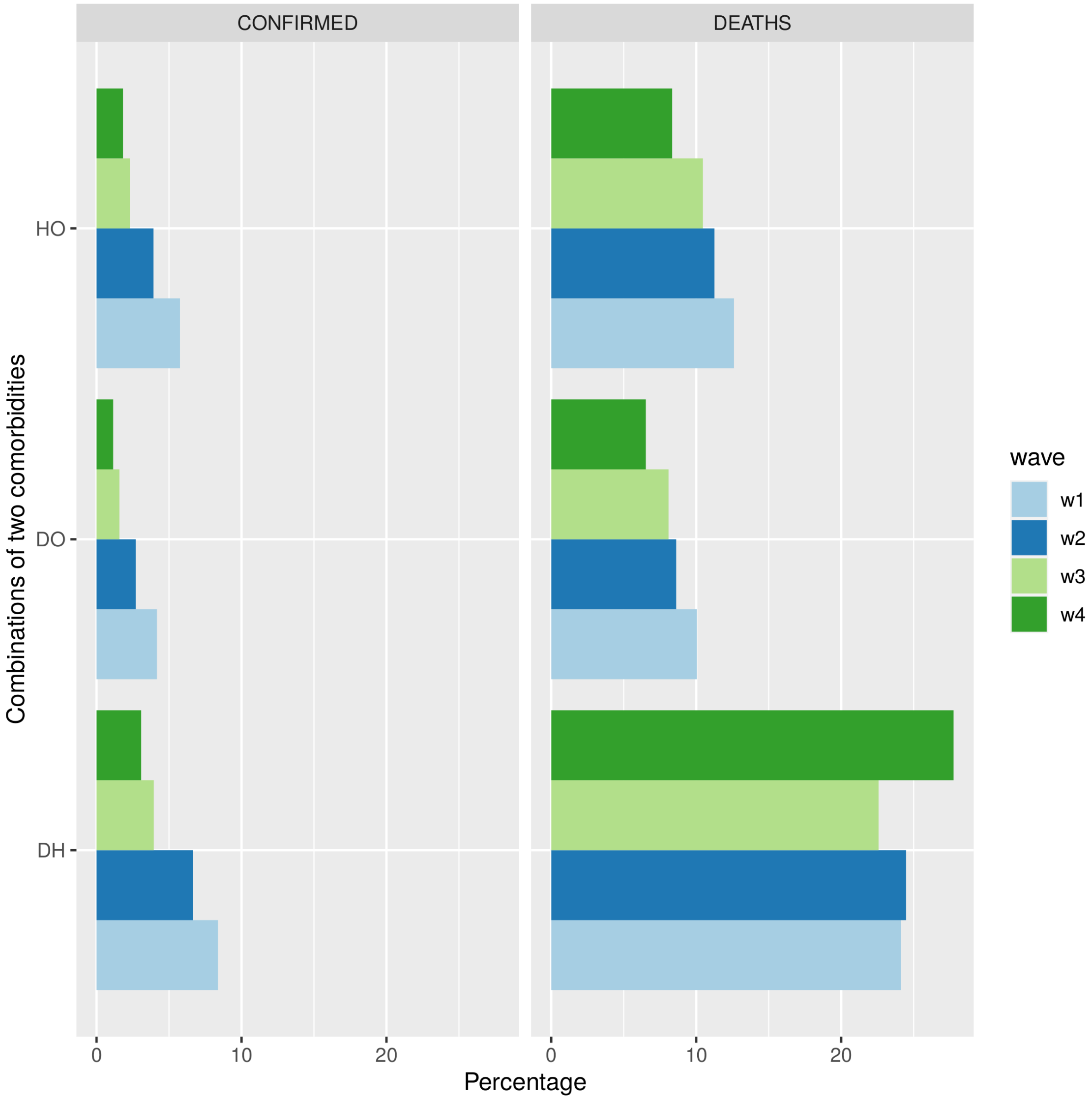

Figure 5 SM. Fraction of the combination of the main comorbidities. The plot presents in the left panel the proportion (%) of CC simultaneously presenting two commodities. The right panel shows the fraction of dead persons who presented two comorbidities. Each combination is denoted with two letters, where "D" stands for diabetes, "H" for hypertension, and "O" for obesity. An example will make the notation clearer: the group with diabetes and hypertension is denoted by "DH."
